# P-1626. Impact of Repeat Blood Cultures Among Adult Cancer Patients with Gram-Negative Bacilli Bacteremia on Patient-Oriented Outcomes

**DOI:** 10.1093/ofid/ofae631.1792

**Published:** 2025-01-29

**Authors:** Wesley Tang, Nancy N Vuong, Ying Jiang, Natalie J Dailey Garnes

**Affiliations:** Baylor College of Medicine, Milwaukie, Oregon; The University of Texas MD Anderson Cancer Center, Houston, Texas; The University of Texas MD Anderson Cancer Center, Houston, Texas; University of Texas MD Anderson Cancer Center, Houston, Texas

## Abstract

**Background:**

Gram-negative bacilli (GNB) bacteremia is a common and often fatal infection with mortality rates estimated to be 14-34%. Follow-up blood cultures (FUBCs) are blood cultures that are repeated after an initial positive culture. FUBCs are used to assess the appropriateness and duration of antimicrobial therapy. Currently, no guidelines exist regarding the use of FUBC for GNB bacteremia, and the utility is not well-studied in adults living with cancer. We aimed to characterize the impact of FUBC in GNB bacteremia on patient-centered outcomes in patients with cancer.

**Methods:**

We conducted a single-center, retrospective study in patients ≥18 years, hospitalized during 2022, living with cancer, and with laboratory-confirmed GNB bacteremia. FUBC was defined as a blood culture performed within 7 days of initial positive blood culture. We gathered information on the source of bacteremia, identified organisms, presence of antimicrobial resistance, and comorbidities. Categorical variables were compared using Chi-square or Fisher’s exact test. Independent predictors of length of hospital stay were obtained via generalized linear model analysis. Logistic regression analysis was used to identify predictors of unplanned hospital readmission and mortality.

**Results:**

Overall 94% (590/626) of patients with GNB bacteremia had a FUBC at our institution. Among those with FUBC, the majority were without growth (73%). 116/590 (20%) of patients had persistent GNB bacteremia. Patients with persistent or alternative growth on FUBC had higher mortality compared to those with negative FUBC (41% and 53% vs 17%, p< 0.001). Patients with a new organism on FUBC had longer length of stay compared to those with no growth on FUBC (aOR 1.55, 95% CI [1.09-2.20]). Patients with a single organism on repeat culture had a higher rate of mortality.

**Conclusion:**

The majority of patients with FUBC did not show growth. We observed that patients who underwent FUBC had lower mortality than those without FUBC. Patients with new organisms identified on FUBC were associated with longer length of stay. Patients with positive FUBC may be characterized as high risk for mortality. Despite the majority of FUBC without growth, there may be a mortality benefit in identifying those patients with positive FUBC in patients living with cancer.

**Disclosures:**

**Natalie J Dailey Garnes, MD, MPH, MS**, AlloVir: Grant/Research Support

